# Where Does Honey Bee (*Apis mellifera* L.) Pollen Come from? A Study of Pollen Collected from Colonies at Ornamental Plant Nurseries

**DOI:** 10.3390/insects13080744

**Published:** 2022-08-18

**Authors:** Kimberly A. Stoner, Andrea Nurse, Robert W. Koethe, Maxwell S. Hatala, David M. Lehmann

**Affiliations:** 1 Connecticut Agricultural Experiment Station, New Haven, CT 06504, USA; 2 Climate Change Institute, University of Maine, Orono, ME 04469, USA; 3 Region 1 Office, Land, Chemicals and Redevelopment Division RCRA, UST and Pesticides Section, U.S. Environmental Protection Agency, Boston, MA 27711, USA; 4 Oak Ridge Associated Universities, Oak Ridge, TN 37830, USA; 5 Center for Public Health and Environmental Assessment, Health and Environmental Effects Assessment Division, Integrated Health Assessment Branch, U.S. Environmental Protection Agency, Research Triangle Park, Durham, NC 27711, USA

**Keywords:** honey bee, *Apis mellifera*, palynology, pollen analysis, pollen foraging, ornamental plant nursery, landscape analysis

## Abstract

**Simple Summary:**

Pollen is the main source of protein, fats, and many micronutrients for honey bees, and it also has the potential to be a major route of exposure to pesticides. The objective of this study was to quantify to what extent honey bee colonies use ornamental nursery plants as sources of pollen over the season. We put honey bee colonies at two large commercial ornamental plant nurseries and used a pollen-trapping device to collect pollen from foraging honey bees as they returned to the hive. Pollen was collected each week from June until September in 2015 and 2018. Samples from the pollen collected were identified to genus by a pollen specialist. By counting and measuring the pollen grains, we could quantify how much of the pollen came from what plant source. We found that most of the pollen in July and August was collected from plant genera not grown at the nursery, including clover (*Trifolium*), maize (*Zea*), buckwheat (*Fagopyrum*), and jewelweed, and related species (*Impatiens*). Genera grown at the nurseries and found in the honey bee-collected pollen in June and early July included roses (*Rosa*), sumac (*Rhus*), and hollies (*Ilex*), but each of these genera also include native or naturalized species that are abundant in the surrounding area, so the pollen probably came from both the nursery and the surroundings.

**Abstract:**

Ornamental nursery plants are both a major agricultural industry in the U.S. and a major feature of the urban and suburban landscape. Interest in their relationship with pollinators is two-fold: the extent to which they provide a nutritional benefit to pollinators, and the extent to which they have the potential to harm pollinators by exposing them to pesticide residues in nectar and pollen. We identified plant genera as sources of trapped pollen collected by honey bee colonies located at commercial ornamental plant nurseries in Connecticut in 2015 and 2018 and quantified the percentage of pollen volume collected from each genus for each weekly sample over two seasons. Plant genera grown at these nurseries, particularly *Rosa*, *Rhus,* and *Ilex*, contributed substantially to pollen volume during weeks 23–27 of the year. Among the genera not grown in nurseries, *Toxicodendron* was also important during weeks 23 and 24, and *Trifolium* was important in both frequency and quantity throughout the season. *Zea* was a major component of pollen volume from weeks 28–36 in both sites, even though cropland was not over 11% of land cover at either site.

## 1. Introduction

In recent years, there has been great interest in the role of ornamental plants in providing resources to bees. Popular books [1], websites [2], scientific research papers [3], and reviews [4] provide information to gardeners looking for flowering plants that are both aesthetically pleasing and pollinator-friendly. Ornamental flowering plants can be highly attractive to diverse pollinator taxa, although visitation varies by genus, species, and even cultivar [3,5,6,7]. On the other hand, systemic pesticides are labeled for use on ornamental plants in the nursery and landscape industries at higher rates than are allowed for other agricultural crops [8], which could result in hazardous exposures to bees in nectar [9] and in pollen [10,11]. Thus, there is a two-fold interest in utilization of ornamental plants by bees: as a food resource and as a possible source of hazardous exposures to pesticides [2].

Most previous reports on attractiveness of ornamental plants to pollinators have focused on measuring visitation [3,4,5,7,12]. In the case of honey bees, floral visitation is mostly a measure of nectar collection, because over 80% of honey bee foraging trips are for nectar [13,14]. Although nectar provides the carbohydrates needed for the colony’s energy and the basis for harvestable honey, pollen is the primary source of essential amino acids, lipids, vitamins, and other micronutrients for the growth and sustenance of honey bee colonies [15].

Pollen trapping provides a method of sampling pollen as it is collected by foraging honey bee workers through the season, making it available for multiple levels of analysis: identification of plant sources of pollen through palynology [16] or molecular methods [17,18], and identifying and quantifying pesticides residues as a measure of pesticide risks [11,19]. This approach has been used to measure pollen collection and pesticide exposure in relation to many agricultural environments: maize and soybean regions in the Midwestern U.S. [20,21], apple orchards in the Northeastern U.S. [22], and mixed intensive agricultural areas in Europe [23,24,25,26].

This paper is one of a series using pollen trapping as a tool for understanding how honey bees relate to the agricultural production of ornamental nursery plants through pollen [11,27]. The production of ornamental nursery plants is a major industry in the U.S., with annual sales of USD 4.545 billion as of 2019 [28], and a major industry in Connecticut, with USD 103.4 million in annual sales as of the 2017 Census of Agriculture [29]. Consumers across the U.S. buying ornamental plants are seeking plants labeled as “pollinator friendly”, and producers also see this as an effective marketing label [30]. This is often framed in terms of reducing the toxicity of pesticide residues in nectar and pollen, but logically should also include whether the plant contributes to bee nutrition.

Previous work from ornamental plant nurseries in Connecticut used DNA metabarcoding for analysis of plant sources of pollen [27]. Although DNA metabarcoding is valuable for identifying plant sources of pollen at the family and genus level [21,31,32], it is not a reliable method for quantification [31,33,34]. In this paper, we have focused on quantification of plant sources of pollen, using the full capacity of palynology to quantify the contribution of plant genera to the pollen by volume across two seasons of pollen collection.

Among the advantages of microscopic analysis of pollen is the ability to quantify the amount of pollen from different plant sources on several levels relevant to understanding the dietary importance of a plant source, using counts and measurements of identified pollen types to calculate pollen volume [26,35,36], which is proportional to pollen weight [37,38]. The objective of this study was to quantify the extent to which honey bees used ornamental nursery plants as sources of pollen and to identify the most important plant genera by percentage of pollen volume used as pollen sources through the season.

## 2. Materials and Methods

*Pollen collection.* Pollen samples were collected in 2015 and 2018 using Sundance bottom-mounted pollen traps (Ross Rounds, Inc. Canandaigua, NY) at two ornamental plant nurseries: Prides Corner Farms, Lebanon, Connecticut (41°36′54″ N, 72°12′52″ W) and Monrovia Nursery, Granby, Connecticut (41°55′55″ N, 72°47′10″ W). Prides Corner Farms covers 168 ha of cultivated area, and Monrovia Nursery is 183 ha. The two sites are 59 km apart.

Detailed descriptions of collection methods used in 2015 are given in Stoner et al. [11] and Sponsler et al. [27]. Pollen samples were collected weekly in 2015 from 28 May to 10 September at Prides Corner Farms and 3 June to 23 September at Monrovia Nursery. Three colonies were located at each nursery, with trapping rotating each week so that pollen was trapped from two colonies at a time, with the trap on the remaining colony set on bypass, and the colony was allowed to retain pollen for its own use. No supplementation with pollen or syrup was provided.

Pollen collection methods used in 2018 differed from those in 2015 in that four honey bee colonies were installed at each site, with two colonies trapping pollen for two weeks, and then set on bypass for two weeks to allow more time for the colony to collect pollen for its own use. Pollen was collected weekly, and colonies were inspected to make sure they were queenright, with queen replacement as needed. Pollen was collected in 2018 from 7 June (Prides Corner Farms) or 8 June (Monrovia Nursery) 2018 to 6 September (both sites) in 2018. Pollen from each hive was collected and stored separately, and frozen in Ziploc quart freezer bags in standard freezers (−18 °C) immediately upon return to the laboratory until use.

*Land cover map and categorization.* Recognizing that honey bees are more likely to forage within a radius of 0.8 km around their hive, land cover characteristics were quantified within this zone and also within a 4 km radius, representing a maximum foraging range [13]. The GPS coordinates for each honey bee deployment site were mapped on ArcGIS Pro (V2.9.2; Esri Inc., Redlands, CA, USA). Concentric rings (i.e., buffers) were plotted on the map around each site. We used the 2019 National Land Cover Database (NLCD) to classify the types of land cover surrounding each site within the two radii [39]. The NLCD classifies land cover into eight different primary categories, including water, developed, barren, forest, shrubland, herbaceous, planted/cultivated, and wetlands [39]. Except for barren land, these classes are each composed of subcategories with unique characteristics. For example, there are four subcategories of developed land (e.g., developed/open space, developed/low, developed/medium, and developed/high intensity). We also summed the percentages of land cover for deciduous forest, evergreen forest, and mixed forest to determine the total percentage of forest, the percentages of shrub/scrub and grassland/herbaceous to determine the total percentage of grassland/herbaceous, and the percentages of land cover for woody wetlands and emergent herbaceous wetlands to determine the total percentage of wetlands. Aerial images of the areas around each site were collected from the ESRI Imagery basemap [40] and extracted using ArcGIS Pro.

*Palynology.* For the 2015 pollen, a single subsample, ranging in size from 0.47 to 0.85 g, from each bulk pollen sample (kept separate by hive and sample date) was sent to the Climate Change Institute, University of Maine, Orono, for microscopy. A total of 43 sub-samples, 21 from Prides Corner Farms, and 22 from Monrovia Nursery in 2015, were analyzed. Selected samples from these sites and one additional nursery were used in Sponsler et al. [27] as a palynological cross-check on DNA metabarcoding results, but here we are using the palynology results from across the entire season at two nurseries in our analysis. The third nursery included in pesticide analysis by Stoner [11] and DNA metabarcoding in Sponsler [27] is not included here because of incomplete records of plant genera grown at the nursery for comparison with the palynological results.

For the 2018 pollen, three subsamples with approximately 0.5 g in each (mean = 0.502, s.d. = 0.085) were processed, mounted on slides, and analyzed separately for each hive and date at the same laboratory. After confirming consistency among subsamples, results from the three subsamples were combined by hive and date for further data analysis. For the 2018 pollen, 24 of these combined hive X date samples from Monrovia Nursery and 26 from Prides Corner Farms were analyzed.

Acetolysis procedures were adapted from Faegri et al. [41]. Pellets were first disarticulated with 10% hydrochloric acid. Glacial acetic acid washes dehydrated the samples before acetolysis. A 9:1 mixture of acetic anhydride and sulfuric acid removed cellular contents and the cellulose wall (intine) to clarify sculptural characters of the pollen exine. The acetolyzed pollen sample was then dehydrated with multiple washes in 95% ethanol and suspended in silicone oil. Samples for pollen analysis were mounted on glass slides under 23 mm^2^ coverslips and examined under light microscopy at 40× magnification. Each slide was scanned in its entirety, and all pollen types present were identified to their plant family, genus, or species with the greatest specificity possible. Pollen grains were counted along marked transects until all pollen species were recorded and a minimum of 300 pollen grains counted per slide (mean = 368, s.d. = 31.0).

Pollen identification followed standard keys [41,42,43,44,45,46,47], and the extensive pollen reference collection at the Climate Change Institute (CCI). This study added over 100 pollen taxa to the CCI pollen reference collection with over half of the reference taxa used coming from Connecticut. In most cases, pollen was identified to genus, but some pollen types could be identified only to family (e.g., some types within the Fabaceae) or were marked as “cf”, which stands for the Latin “confer” or “conferatur”, both meaning “compare”. This means the genus given is the closest match to the reference specimens or literature references available, but the identification is not entirely certain. Pairs of closely related genera, such as *Eupatorium* and *Eutrochium* or *Dasiphora* and *Potentilla,* are listed together because they cannot be reliably separated. Common and Latin names used in the text, Appendix A Table A1, and Supplementary Materials follow Haines [48].

*Calculations of volume of pollen by plant source.* We calculated the pollen volume of each pollen type (identified by family, genus, or species) in each trapped pollen sample collected over two years at both sites. To calculate the volume of each pollen type, we measured the length of the polar and equatorial axes of typical grains of each taxon. The volume per pollen grain of each pollen taxon was calculated (Appendix A Table A1) based on formulae for different pollen shapes (spherical, prolate, or oblate) [35], and then volumes for each taxon were calculated as a percentage of the total pollen volume for the sample [26,35,36,38], using this equation:

$$\begin{array}{l} Percentage\;of\;pollen\;volume\;by\;genus\\ \;=100\times \left(count\;of\;pollen\;grains\times volume\;of\;pollen\;grains\right)\\ \;÷Sum\;of\;total\;pollen\;volume\;for\;all\;taxa \end{array}$$

*Identification of genera grown at each nursery.* Lists of genera for each nursery were compiled from nursery sales, shipping, and pest management records, and from visual observations and discussions with nursery staff (Appendix A Table A2). Records from 2015 and 2018 were combined.

*Statistical methods*. The pollen volume for the genera grown at the nursery where the honey bee colony was located was summed, and the percentage of pollen volume attributed to genera grown at the nursery in relation to the total pollen volume for the sample was calculated for each hive and sample date. The percentage of pollen volume from genera grown at the nursery where the hive was located is presented graphically with descriptive nonparametric statistics using the “boxplot” function in ggplot2 showing the median, 25th, and 75th percentile ranges for each week of the year [49].

## 3. Results

### 3.1. Land Cover Composition at the Nursery Sites

**Figure 1:**
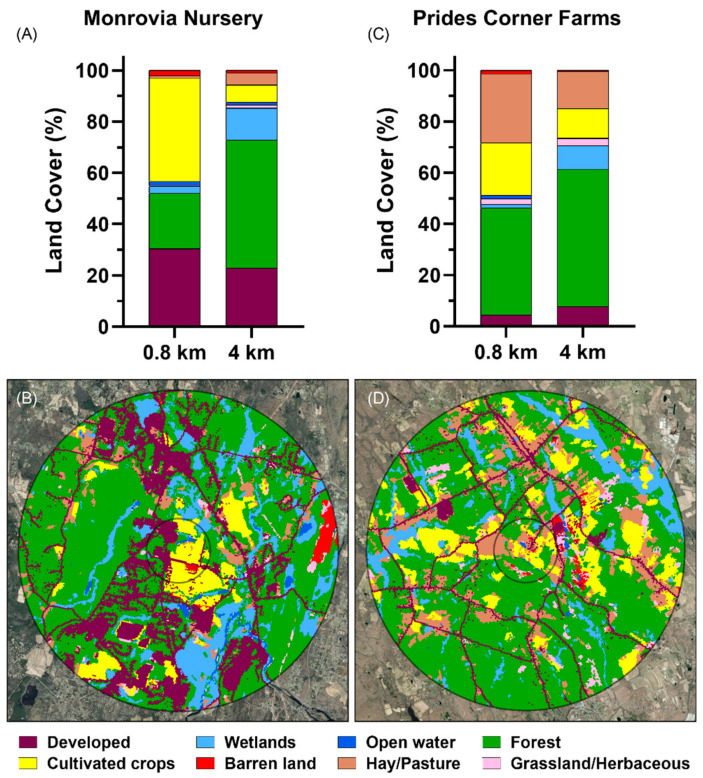
Land cover composition. Land cover composition of the environment surrounding honey bee colonies located at commercial plant nurseries in Connecticut using a 0.8 and 4 km radius around each site. (**A**) Land cover composition expressed as percentages for Monrovia Nursery. (**B**) Aerial photo (scale = 1:55,396) of the environment surrounding the honey bee colonies at Monrovia Nursery. Black rings represent 0.8- and 4 km honey bee foraging radii around each commercial plant nursery. (**C**) Land cover composition for Prides Corner Farms. (**D**) Aerial photo for Prides Corner Farms with land cover composition color-coded within the two foraging radii.

Using the location of the honey bee deployment sites and the NLCD land cover composition, we calculated the percentage of land covered by each category within the radii of 0.8 km and 4 km (Figure 1). Land cover characteristics differed between the two sites, primarily within the 0.8 km radius. Monrovia Nursery had both more developed land (30%) and more land in cultivation (40%) than Prides Corner Farms within the same area (4.5% and 20%, respectively). Prides Corner Farms had more pasture/hay (27%) and forest (42%) within the 0.8 km radius than Monrovia Nursery (0.9% and 22%, respectively). On a wider scale, Monrovia Nursery is in a more suburban environment, with 23% of the land developed within a 4 km radius, compared to 8% for Prides Corner Farms, which is in more rural surroundings. Both nurseries have relatively little cultivated cropland in the wider surroundings, 7% at Monrovia Nursery and 11% at Prides Corner Farms, with forested land dominating at the wider scale at both sites (50% at Monrovia Nursery, 54% at Prides Corner Farms).

### 3.2. Percent of Total Pollen Volume from Genera Grown at the Nursery

**Figure 2:**
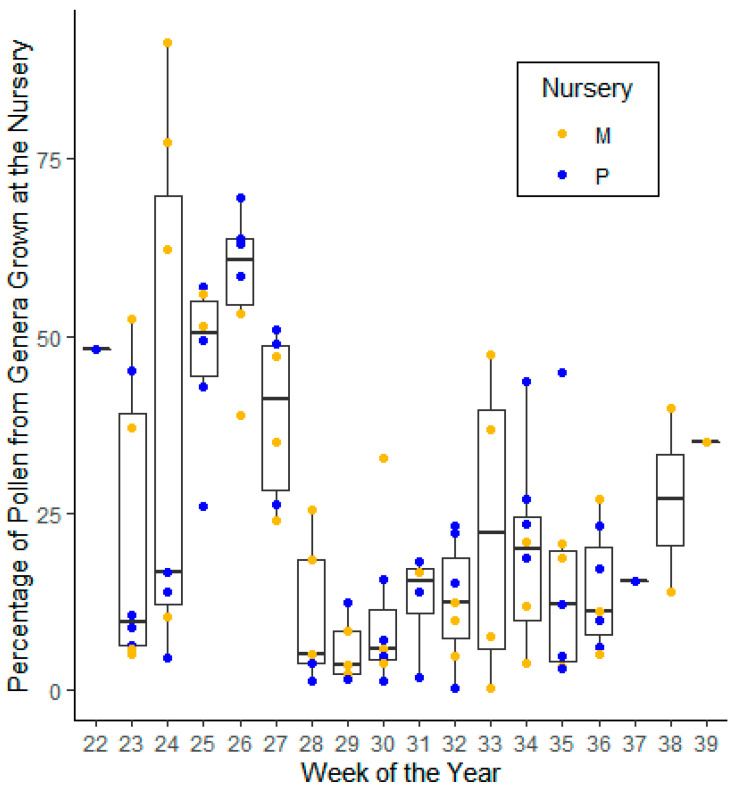
Percentage of pollen volume in each sample coming from a plant genus grown at the nursery where the hive was located. M = Monrovia Nursery, P = Prides Corner Farms. Colored points show results of individual samples, including those from both 2015 and 2018. Boxplot shows median, 25th percentile, 75th percentile, and lines within 1.5 × the interquartile distance for each week of the season of pollen trapping (varying with site and year but extending from 28 May to 23 September).

For each trapped pollen sample from the two sites and two years of trapping, we calculated pollen volume for each taxon and the percentage of the total pollen volume represented by each genus as described above. Then, we summed the percentage of total pollen volume for those genera grown at the nursery, as shown in Figure 2. This percentage (or proportion) represents the maximum amount of the pollen that could have come from the nursery, because honey bees forage over an area larger than the nursery, and often the genera grown at the nursery, such as *Rosa* and *Rhus*, include species that grow wild in the area.

We found that the percentage of pollen volume that could have come from the ornamental plants grown at the nurseries was highly variable in weeks 23–24 during early to mid-June (Figure 2). Week 24 was especially variable with three samples from Monrovia Nursery at 62%, 77%, and 92% from genera grown at the nurseries, and the remaining samples all below 17%. Weeks 25 and 26 had generally high proportions of pollen volume from genera at the nurseries, with medians above 50%. The proportion of pollen from genera grown at the nurseries began dropping in week 27, and then remained low, with medians below 25%, until weeks 38 and 39, when the proportions increased in the two of the three samples taken at Monrovia Nursery at the end of the season in 2015.

### 3.3. Genera in Pollen Samples at Each Nursery

**Figure 3:**
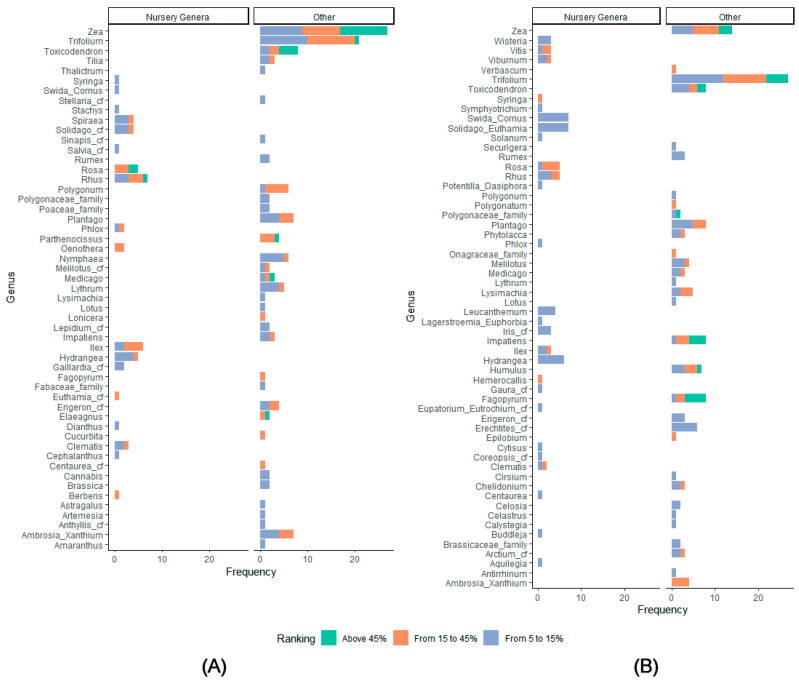
Frequency of identification of pollen genera at ≥5% of a sample by volume. Color of the bar indicates the percentage of pollen volume represented by a particular genus for that sample based on the calculation of pollen volume (see methods for further explanation). (**A**) Pollen trapped from Monrovia Nursery in 2015 and 2018 out of 46 total samples; (**B**) Pollen trapped from Prides Corner Farms in 2015 and 2018 out of 47 total samples.

Most of the genera occurring in the pollen samples at a minimum of 5% of the pollen volume were not grown at either nursery (Figure 3). Figure 3 presents the frequency of occurrence for each genus at different levels: 5 to 15%, 15 to 45%, or greater than 45%. At both sites, *Zea* (maize), *Trifolium* (several species of clover), and *Toxicodendron* (poison ivy and poison sumac), none of which are ornamental nursery plants, were major sources of pollen, occurring frequently above 5% of the pollen volume, and occasionally above 45% of the pollen volume. The genera in the pollen samples at each site and their frequency of occurrence at these levels are described below.

**Monrovia Nursery (Figure 3A).** At Monrovia Nursery, *Zea* was the genus most frequently found at a volume ≥5%, with 10 samples over 45%, 8 samples from 15 to 45%, and 9 samples from 5 to 15%, for a total of 27 samples ≥5% out of a total of 46 samples analyzed. *Trifolium* followed with 1 sample over 45%, 10 samples from 15 to 45%, and 10 samples from 5 to 15%, for a total of 21 ≥5% out of 46 samples. *Toxicodendron* was also found both frequently and in high proportions, with 4 samples above 45%, 2 samples from 15 to 45%, and 2 samples from 5 to 15% for a total of 8 ≥5% out of 46 samples. Other pollen types not grown at the nursery but frequently found at ≥5% of pollen volume were: *Ambrosia/Xanthium* (ragweed and cocklebur), 7 samples; *Plantago* (plantain), 7 samples; and *Polygonum* (knotweed) and *Nymphaea* (water lily) with 6 samples each. Other pollen types not grown at Monrovia Nursery but with at least 45% in one sample were *Parthenocissus* (Virginia creeper and Boston ivy), *Medicago* (medick and alfalfa), and *Eleagnus* (autumn-olive and Russian-olive).

Among the genera actually grown at Monrovia Nursery, only *Rosa* (rose) and *Rhus* (sumac) ever composed more than 45% of any sample (2 samples for *Rosa* and 1 for *Rhus*). *Rhus* was found at ≥5% in 7 samples, *Ilex* (holly) in 6 samples, and *Rosa* and *Hydrangea* (hydrangea) in 5 samples.

**Prides Corner Farms (Figure 3B).** *Trifolium* was most frequently found ≥5%, with 5 samples above 45%, 10 samples from 15 to 45%, and 12 samples from 5 to 15%, for a total of 27 samples ≥5% of pollen volume out of a total of 47 samples analyzed at this site. *Zea* followed, with 3 samples above 45%, 6 from 15 to 45%, and 5 from 5 to 15%, totaling 14 samples ≥5%. *Toxicodendron*, *Impatiens* (touch-me-not), *Fagopyrum* (buckwheat), and *Plantago* all had 8 samples ≥5% of pollen volume, with the first three also including samples above 45%. *Humulus* (hop) had 7 samples ≥5% of pollen volume, with one above 45%.

No genus grown at Prides Corner Farms ever composed more than 45% of any pollen sample. Genera grown at Prides Corner Farms that were found most frequently above 5% of pollen volume were *Swida/Cornus* (dogwood) and *Solidago/Euthamia* (goldenrod), both with 7 samples, all from 5 to 15%, followed by *Hydrangea*, with 6 samples. *Rosa* and *Rhus* both had 5 samples ≥5% of pollen volume.

### 3.4. Major Plant Sources of Pollen through the Season

**Figure 4:**
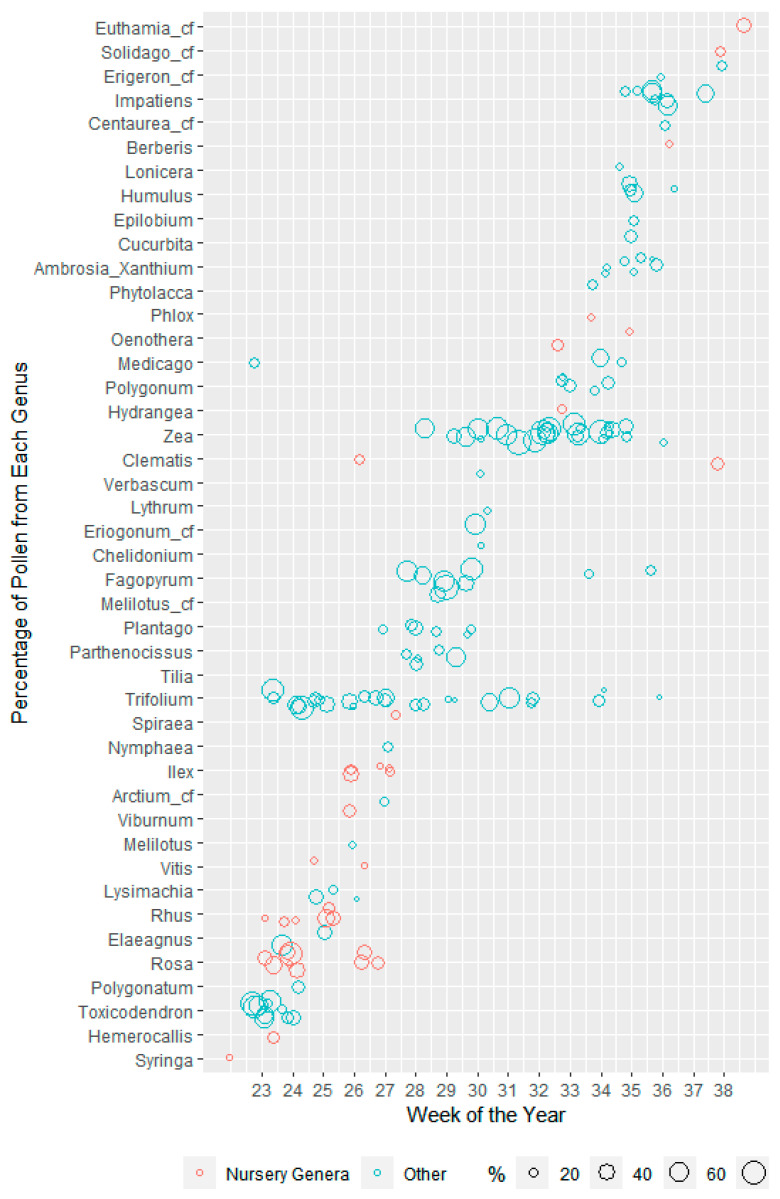
Major sources of pollen through the season by genus. Each bubble represents a genus that represented a percentage of at least 15% of the pollen in a sample trapped from a honey bee colony, with the size of the bubble representing the percentage that genus represented from the total volume of the pollen sample, and the color indicating whether the genus was grown at the nursery. This figure includes pollen samples from both Monrovia Nursery and Prides Corner Farms from both 2015 and 2018. Note that jittering was used to make bubbles visible for the same genus for multiple samples in the same week, slightly altering the alignment with the grid.

In Figure 4, the major sources of pollen were narrowed down further to those that composed a proportion of at least 15% of pollen volume in at least one sample and then graphed across the pollen trapping season.

**Seasonality of Genera Grown at the Nurseries as Major Pollen Sources.** Genera grown at the nurseries primarily contributed to the trapped pollen early in the season (weeks 22–27), with *Rosa*, *Rhus,* and *Ilex* concentrated during those weeks. *Rosa* was a prominent component of two samples from week 23 (35% and 51% of the samples) and 24, then trailed off after week 27, with *Rhus* starting in week 23 and peaking at week 25 with 3 samples (26%, 34%, and 52%). *Ilex* then peaked at week 26 (24% and 44%) and continued through week 27. Later in the season, *Hydrangea* and *Oenothera* (evening-primrose and bee-blossom) together contributed to the higher proportion of pollen from nursery genera in two samples from Monrovia Nursery in week 33, and *Clematis* (virgin’s-bower) along with *Solidago* (goldenrod) in week 38 and *Euthamia* (grass-leaved goldenrod) in week 39.

**Seasonality of Genera Not Grown at the Nurseries as Major Pollen Sources.***Toxicodendron* was a major pollen source (from 46% to 82%) in six samples in week 23, continuing into week 24. *Trifolium* was also a major pollen source beginning in week 23 (27% and 76%), and peaked in week 24 (34%, 49%, and 90%), but also continued through week 32, with a few smaller proportions in later weeks. *Fagopyrum* was a major pollen source beginning in week 28 (69% and 52%) and continued through week 29 (69% and 95%) and 30 (41% and 79%). *Zea* was a major source starting with one sample each in weeks 28 (56%) and 29 (36%) and continued with multiple samples per week with proportions ranging from 19 to 96% through week 35. *Impatiens* was a major source late in the season, particularly in week 36, with 4 samples ranging from 22 to 55%.

## 4. Discussion

Using pollen trapping and microscopy-based palynology, we identified and quantified the floral sources of pollen collected by honey bee foragers in two ornamental plant nurseries located in Connecticut. Overall, most of the contribution to honey bee pollen of genera grown at the nurseries was during the early weeks of pollen trapping, up through week 27 (all of June and the first week of July), particularly at Monrovia Nursery (Figure 2). This was in large part due to *Rosa* and *Rhus*, each of which supplied the majority of the pollen volume in multiple samples at Monrovia Farms—in one sample, *Rosa* was 89% of the pollen volume.

It should be kept in mind that percentages given throughout for genera grown at the nurseries are the maximum that could have come from inside the nursery. For example, while *Rosa* made up a significant percentage of annual plant sales at both nurseries (4.3% at Prides Corner Farms and 10.4% at Monrovia Nursery, Appendix A Table A2), there are also 16 species of *Rosa* recorded as occurring in the wild in Connecticut [48], including the invasive species *Rosa multiflora* Thunb., which is abundant in pastures, field edges, and along roadsides [50]. Both cultivated roses and multiflora rose bloom in Connecticut in early June, when rose pollen was a major component of the trapped pollen (personal observation, K.A.S.). *Rhus*, another genus grown at the nurseries and contributing to trapped pollen, is a minor crop at both nurseries, but includes 4 native species in Connecticut and is abundant growing wild along roadsides, forest edges, and dry fields [48].

Other early summer contributors to pollen from genera grown at the nurseries were *Ilex*, at both nurseries; *Syringa, Hemerocallis, Vitis, Viburnum,* and *Clematis* at Prides Corner Farms; and *Spiraea* at Monrovia Nursery. *Spiraea* is of particular interest because previous research found high levels of pesticides associated with *Spiraea* pollen at another ornamental plant nursery in Connecticut, not included here [11]. We found *Spiraea* pollen above 5% of the pollen volume in only 4 samples, all at Monrovia Nursery (Figure 3A), with only a single sample above 15% (Figure 3A and Figure 4).

Of the genera not grown at the nurseries, *Trifolium* was a major source across much of the season and was also the most frequently occurring pollen source in a previous study that included our sites [27]. The family Fabaceae, to which *Trifolium* belongs, was found to be a major pollen source in several studies in the Northeast and Midwest of the U.S. using a variety of techniques for pollen identification and quantification [16,21,32].

A surprise finding was that *Zea* was a major pollen source in both quantity and frequency starting in late July and continuing to late August at both sites, which has not been a common finding in the United States. In several studies conducted in intensively agricultural regions of the Midwestern U.S., where maize is a major component of the landscape, honey bees collected little or no maize pollen [21,51,52], although Krupke et al. [53] was an exception.

In contrast, several studies conducted in Europe found that maize was a major source of pollen, even when maize occupied only a small proportion (as little as 4%) of the surrounding landscape [24,26,38]. In an analysis of 114 datasets, mainly from Europe, Keller et al. [54] found that maize was among the 5 most common pollen sources in over 50% of the studies. Recognizing the significance of these findings, additional studies conducted in Europe evaluated the effects of maize pollen on honey bee health. Results showed that a diet of maize pollen has been shown to reduce honey bee longevity and brood production [55], and mixed pollen with a high proportion of maize (70%) negatively affected honey bee survival, hypopharyngeal gland development, and vitellogenin production [56].

In the nursery sites studied here, cropland of all kinds (including the nurseries themselves) occupied only 7 to 11% of the land cover in the 4 km foraging area, but in 10 samples at Monrovia Nursery and 3 samples at Prides Corner Farms, *Zea* represented over 45% of pollen volume (Figure 3). Part of the variation in overall results may stem from variability in pollen foraging behavior among colonies, even in the same site in the same year. One colony at Prides Corner Farms collected 92% and 96% maize pollen over two weeks, and also collected an unusually large amount of pollen (523 g and 1495 g), so that maize pollen represented 49% of all the trapped pollen for that colony over the season (P8, Supplementary Materials), while the other colony over the same two weeks collected 64% and 45% maize pollen, but in much smaller samples (121 g and 161 g), so that maize represented only 5% of the pollen for the season (P6, Supplementary Materials).

Because this study was based on pollen trapped from honey bee colonies, we can address only the extent to which these ornamental plant genera provide pollen to honey bees, rather than to the wide diversity of wild bees and other pollinators. As has been recognized for over a century, bees vary widely in their pollen utilization, from specialization on just a few closely related species to broad generalization, with many different foraging strategies and pollen-collecting behaviors [57,58].

Consumers across the U.S. are seeking “pollinator friendly” ornamental plants (Khachatryan et al. 2020). Our results show that overall, genera grown at the nurseries constitute only a part of the rich diversity of pollen sources available to honey bee colonies early in the summer, along with non-nursery genera like *Trifolium*, *Toxicodendron*, and *Eleagnus*. Dependence on nursery-grown genera drops off sharply as a component of pollen collections after mid-July (week 28), and the honey bees relied much more heavily on a mixture of crops (*Zea*, *Fagopyrum, Medicago, Humulus*), and herbaceous weeds and wildflowers (*Trifolium, Impatiens*), for the rest of the summer. These findings suggest that honey bee colonies do not rely heavily on ornamental plants as sources of pollen, even when they are located in the heart of commercial plant nurseries.

## Data Availability

The data presented in this study are available in article and Supplementary Material.

## Supplementary Materials

The following supporting information can be downloaded at: https://www.mdpi.com/article/10.3390/insects13080744/s1, Figure S1: Weight of trapped pollen by genus over the season for each hive in 2018. Table S1: Weight of trapped pollen by genus over the season for each hive in 2018. Table S2: Weight of pollen collected by location, hive, and date in 2018.

## Disclaimer

This article has been reviewed by the U.S. Environmental Protection Agency and approved for publication. Approval does not signify that the contents necessarily reflect the views and policies of the Agency or of the U.S. Federal Government, nor does the mention of trade names or commercial products constitute endorsement or recommendations for use of those products. The authors report no financial or other conflicts of interest. The authors alone are responsible for the content and writing of this article.

## Appendix A

**Table A1:** Pollen Grain Volumes used in calculations of pollen volume. In general, these volumes are calculated from pollen grains measured for each genus directly from the pollen samples. However, for some genera, such as *Trifolium*, where multiple species were identified with different grain volumes, the volume used was a weighted average of the measured volume for each species. cf = “confer” or “conferatur”, from Latin, both meaning “compare”.

Monrovia Nursery			Prides Corner Farms		
Genus or Family	cf	Grain volume (µm^3^)	Genus or family	cf	Grain volume (µm^3^)
*Acer*		4920	*Achillea*	cf	7238
*Amaranthus*		6371	*Actaea*		22,449
*Ambrosia/Xanthium*		3054	*Aesculus*		3351
*Amorpha*		3393	*Ajuga*		4581
*Andropogon*		22,093	*Alisma*	cf	22,449
*Anthyllis*	cf	8143	*Allium*		6447
*Antirrhinum*		4618	*Ambrosia/Xanthium*		3236
*Aquilegia*		3054	*Amorpha*		3393
*Asparagus*		2360	*Anthyllis*		10,619
*Astragalus*		5236	*Aquilegia*		4817
*Baptisia*		2121	*Aralia*		11,494
*Bellis*	cf	8181	*Arctium*	cf	3054
*Berberis*		22,449	*Asparagus*		2360
*Calystegia*		203,689	*Astragalus*	cf	1595
*Capsella*	cf	1767	*Baptisia*		1227
*Carum*	cf	1697	*Begonia*		295
*Castanea*		636	*Bellis*	cf	8181
*Centaurea*		11,494	*Buddleja*		733
*Cephalanthus*		3393	*Buxus*	cf	14,137
*Chelidonium*		6371	*Callitriche*		697
*Chenopodium*		9203	*Campanula*		11,494
*Cirsium*		22,449	*Capsella*	cf	1767
*Clematis*		8181	*Caragana*		1593
*Coreopsis*		11,494	*Cardamine*	cf	3054
*Cucumis*		87,114	*Carya*		23,732
*Cucurbita*		530,241	*Castanea*		636
*Daucus*	cf	1882	*Celastrus*		6635
*Dianthus*	cf	47,713	*Celosia*		28,731
*Epilobium*	cf	523,600	*Centaurea*		11,494
*Erigeron*	cf	6371	*Cephalanthus*		3393
*Eupatorium/Eutrochium*		6648	*Chelidonium*		11,494
*Euphorbia*		8310	*Chenopodium*		6371
*Fagopyrum*		7238	*Cichorium*	cf	47,713
*Fallopia*		3393	*Cirsium*		22,449
*Fraxinus*		4189	*Clematis*		5540
*Fuchsia*	cf	102,161	*Colutea*	cf	3485
*Funaria*	moss	2124	*Coreopsis*		11,494
*Gaillardia*	cf	41,630	*Crocosmia*	cf	17,999
*Geranium*		77,952	*Cytisus*		3732
*Hedera*	cf	17,157	*Dahlia*	cf	15,599
*Heiracium*		7238	*Daucus*	cf	1882
*Helianthus*	cf	18,817	*Digitalis*		4189
*Hemerocallis*		44,899	*Echinops*		25,656
*Heuchera*		905	*Epilobium*		747,596
*Hydrangea*		1327	*Erigeron*	cf	6086
*Hypericum*		509	*Eupatorium/Eutrochium*	cf	4398
*Ilex*		10,688	*Fagopyrum*		32,071
*Impatiens*		18,817	*Fagus*		7202
*Iris*		59,362	*Funaria*	moss	1947
*Lepidium*	cf	8181	*Gaillardia*		41,630
*Liatris*	cf	11,494	*Galium*		2572
*Linaria*		2547	*Gaura*	cf	696,912
*Lotus*		1327	*Heiracium*	cf	7238
*Lycium*		14,137	*Helianthus*		18,817
*Lysimachia*		5445	*Hemerocallis*		73,999
*Lythrum*		11,579	*Hippuris*		8181
*Medicago*		8585	*Humulus*		6371
*Melilotus*	cf	4920	*Hydrangea*		1327
*Mentha*		25,656	*Hypericum*		509
*Nuphar*		17,974	*Ilex*		8181
*Nymphaea*		17,974	*Impatiens*		6283
*Paeonia*		10,263	*Iris*		35,278
*Parthenocissus*		13,932	*Lagerstroemia*		15,080
*Philadelphus*		1593	*Lepidium*		324
*Phlox*		47,713	*Lespedeza*		2681
*Phryma*		4817	*Liatris*	cf	11,494
*Phytolacca*		10,263	*Lamium*	cf	9140
*Pinus*		78,703	*Liquidambar*		28,731
*Plantago*		7588	*Lonicera*	cf	38,725
*Polygonum*		11,494	*Lotus*		1327
*Pontederia*		6049	*Lupinus*		3563
*Potentilla/Dasiphora*		1593	*Lysimachia*		2356
*Primula*		11,451	*Lythrum*		8890
*Quercus*		6648	*Malus*	cf	6925
*Raphanus*	cf	3902	*Medicago*		5052
*Rhamnus*		3223	*Melilotus*		4920
*Rhododendron*		32,511	*Mikania*	cf	14,137
*Rhus*		13,854	*Morus*		1767
*Robinia*		6097	*Myriophyllum*		41,630
*Rosa*		5231	*Nyssa*		13,547
*Rubus*		2686	*Onobrychis*		5089
*Rumex*		8084	*Pedicularis*		1327
*Sagitaria*		6371	*Persicaria*		33,510
*Salvia*		16,605	*Phlox*		47,713
*Saxifraga*		4189	*Plantago*		7156
*Scutellaria*		8181	*Polygonatum*		56,968
*Solanum*		2015	*Polygonum*		34,024
*Solidago*	cf	3393	*Portulaca*		33,510
*Spergula*		7238	*Potentilla*		1593
*Spiraea*		530	*Potentilla/Dasiphora*		1593
*Stellaria*	cf	7238	*Primula*		637
*Swida*		17,652	*Quercus*		6648
*Symphyotrichum*	cf	14,137	*Ranunculus*		8181
*Syringa*		9193	*Rhamnus*		5195
*Tanacetum*	cf	15,551	*Rhus*		14,380
*Taraxacum*	cf	18,697	*Robinia*		6097
*Tilia*		18,817	*Rosa*		3979
*Toxicodendron*		5753	*Rubus*		6336
*Tragopogon*		65,450	*Rudbeckia*	cf	14,137
*Trifolium summed*		4337	*Rumex*		8818
*Urtica*		4849	*Salvia*		11,494
*Verbascum*		4817	*Sambucus*		3223
*Viburnum*		8181	*Sedum*		3054
*Vitis*		5175	*Solanum*		2145
*Zea*		248,475	*Solidago*	cf	3393
*Zinnia*	cf	47,713	*Sparganium*		11,494
Crassulaceae		2356	*Spiraea*		530
Brassicaceae ≤ 20 µm		3054	*Stellaria/Cerastium*		18,817
			*Swida*		21,069
			*Syringa*		9193
			*Symphoricarpus*		33,510
			*Symphyotrichum*		14,137
			*Taraxacum*	cf	11,494
			*Taxus*		14,137
			*Thalictrum*		4189
			*Toxicodendron*		3817
			*Trifolium summed*		5429
			*Urtica*		1767
			*Verbascum*		4817
			*Veronica*		8033
			*Viburnum*		12,464
			*Viola*		12,315
			*Vitis*		4817
			*Weigela*	cf	47,713
			*Wisteria*		5236
			*Zea*		248,475
			Apiaceae—no genus		1697
			Boraginaceae—no genus		5575
			Brassicaceae—no genus 18–20 um		3054
			Brassicaceae—no genus 20 um		4189
			Crassulaceae—no genus		2356
			Poaceae—large pore no genus		21,167
			Poaceae sp. 2 no genus		19,957
			Poaceae—no genus		4189
			Caprifoliaceae—no genus		4189
			Solanaceae		8181

**Table A2:** List of Flowering Plant Genera at the Nurseries. The initial list of the major plant genera by plant sales (listed in all capital letters with numbers of plants and percentages) was collected by Dr. Richard Cowles at the beginning of the 2015 growing season. This initial list was supplemented with lists from visual surveys at the sites in 2015 and 2018, plant inventories from Monrovia Nursery, and records of plant genera treated from nursery pesticide records.

	Annual Plant Sales as Reported by Nurseries at the Beginning of 2015		Percentages Based on Reported Annual Sales		Additional Cultivated Genera from Visual Surveys, Pesticide Records, or Inventories	
Genus	No. Plants Prides Corner Farms	No. Plants Monrovia Nursery	% Prides Corner Farms	% Monrovia Nursery	Prides Corner Farms	Monrovia Nursery
*Abelia*					X	
*ACER*	33,511	3080	1.4%	0.2%		
*Achillea*					X	X
*Agastache*					X	X
*Ajuga*					X	X
*Allium*						X
*Anemone*					X	X
*Aquilegia*					X	X
*Armeria*						X
*Asclepias*					X	X
*Astilbe*					X	X
*BERBERIS*	17,001	33,693	0.7%	2.2%		
*BUDDLEIA*	33,688	14,634	1.4%	0.9%		
*BUXUS*	129,842	49,348	5.3%	3.2%		
*Calluna*					X	
*Campanula*					X	X
*Caragana*						X
*Caryopteris*					X	X
*Catalpa*						X
*Centaurea*					X	
*Cephalanthus*					X	X
*Cercis*					X	X
*Chaenomeles*					X	X
*CLEMATIS*	22,327	17,975	0.9%	1.2%		
*CLETHRA*	17,583	9380	0.7%	0.6%		
*COREOPSIS*	18,477	13,805	0.8%	0.9%		
*CORNUS* (or *Swida*)	45,303	7128	1.9%	0.5%		
*Cotinus*					X	X
*CYTISUS*	17,892	4827	0.7%	0.3%		
*Delosperma*					X	X
*Delphinium*					X	
*DEUTZIA*	24,336	5103	1.0%	0.3%		
*DIANTHUS*	14,384	2520	0.6%	0.2%		
*Dicentra*						X
*Diervilla*					X	X
*ECHINACEA*	52,876	25,706	2.2%	1.6%		
*Erica*					X	
*EUONYMUS*	57,775	15,331	2.4%	1.0%		
*Eupatorium*					X	X
*FORSYTHIA*	17,852	8016	0.7%	0.5%		
*Fothergilla*					X	X
*Fragaria*					X	X
*Gaillardia*					X	X
*Gaura*					X	X
*Geranium*					X	X
*Geum*					X	X
*Helenium*					X	X
*Heliopsis*					X	X
*HELLEBORUS*	23,545	10,571	1.0%	0.7%		
*HEMEROCALLIS*	62,301	20,352	2.6%	1.3%		
*HEUCHERA*	26,989	22,329	1.1%	1.4%		
*HIBISCUS*	38,486	7057	1.6%	0.5%		
*HOSTA*	54,946	68,531	2.3%	4.4%		
*HYDRANGEA*	247,037	185,588	10.2%	11.9%		
*Hypericum*					X	X
*Iberis*					X	X
*ILEX*	115,836	45,688	4.8%	2.9%		
*IRIS*	17,962	6820	0.7%	0.4%		
*ITEA*	9571	3751	0.4%	0.2%		
*Lagerstroemia*					X	
*KALMIA*	44,291	20,804	1.8%	1.3%		
*Kniphofia*					X	X
*Lamium*						X
*LAVANDULA*	33,734	5532	1.4%	0.4%		
*LEUCANTHEMUM*	15,193	7628	0.6%	0.5%		
*LEUCOTHOE*	24,502	1898	1.0%	0.1%		
*Ligularia*					X	X
*Lobelia*					X	X
*Lonicera*					X	X
*LUPINUS*	18,224		0.7%			
*Lycium*					X	X
*MALUS*	7790	6311	0.3%	0.4%		
*MONARDA*	14,250	2062	0.6%	0.1%		
*NEPETA*	18,642		0.8%			X
*Oenothera*						X
*PAEONIA*	8680	25,522	0.4%	1.6%		
*Penstemon*					X	x
*Perovskia*					X	X
*Philadelphus*					X	
*PHLOX*	94,604	14,683	3.9%	0.9%		
*PHYSOCARPUS*	27,417	8788	1.1%	0.6%		
*PIERIS*	87,271	44,340	3.6%	2.8%		
*Platycodon*					X	X
*POTENTILLA* (or *Dasiphora*)	22,249	6694	0.9%	0.4%		
*PRUNUS*	19,732	22,455	0.8%	1.4%		
*Pyrus*					X	
*RHODODENDRON*	331,757	445,573	13.6%	28.6%		
*Rhus*					X	X
*ROSA*	104,066	162,585	4.3%	10.4%		
*ROSMARINUS*	16,460		0.7%			
*RUBUS*	17,575	9975	0.7%	0.6%		
*RUDBECKIA*	20,905	3597	0.9%	0.2%		
*SALIX*	14,478		0.6%			X
*SALVIA*	26,184	15,425	1.1%	1.0%		
*Sambucus*					X	X
*SEDUM*	16,552	24,497	0.7%	1.6%		
*Solanum* (*Eggplant*)					X	
*Solidago*					X	X
*Sorbaria*					X	X
*SPIRAEA*	80,128	42,086	3.3%	2.7%		
*Stachys*						X
*Stokesia*					X	X
*Symphyotrichum* (*Aster*)					X	X
*Symphoricarpos*					X	
*SYRINGA*	105,496	33,502	4.3%	2.1%		
*Tiarella*					X	X
*VACCINIUM*	49,533	20,200	2.0%	1.3%		
*Veronica*					X	X
*VIBURNUM*	45,960	14,009	1.9%	0.9%		
*VINCA*	17,897	5506	0.7%	0.4%		
*Vitis*					X	X
*WEIGELA*	47,699	35,225	2.0%	2.3%		
total	2,430,789	1,560,130				
