# Supplementary material for: Where Does Honey Bee (Apis mellifera L.) Pollen Come from? A Study of Pollen Collected from Colonies at Ornamental Plant Nurseries
Source: Insects. 2022 Aug 18;13(8):744. doi: 10.3390/insects13080744 (PMC9409349; doi:10.3390/insects13080744)
Supplement: Supplementary file 1 [file insects-13-00744-s001.zip › insects-1815356-supplementary.pdf]

insects

MDPI

Supplemental Figure S1

Methods:

Calculation of weight of pollen from each plant genus. In 2018, pollen collected from each colony each week was weighed (which was not done in 2015), and varied widely (mean = 339 g, s.d. = 372 g, range 4.92 g to 1582 g). Assuming that pollen weight is proportional to volume [Odoux et al 2012], the proportion by volume and thus weight of each taxon was then applied to the total weight of the pollen collected from that hive and sample date, and summed over the samples from that hive over the season [35], which allowed us to calculate a profile of the pollen collected over the season. s.d. = standard deviation.

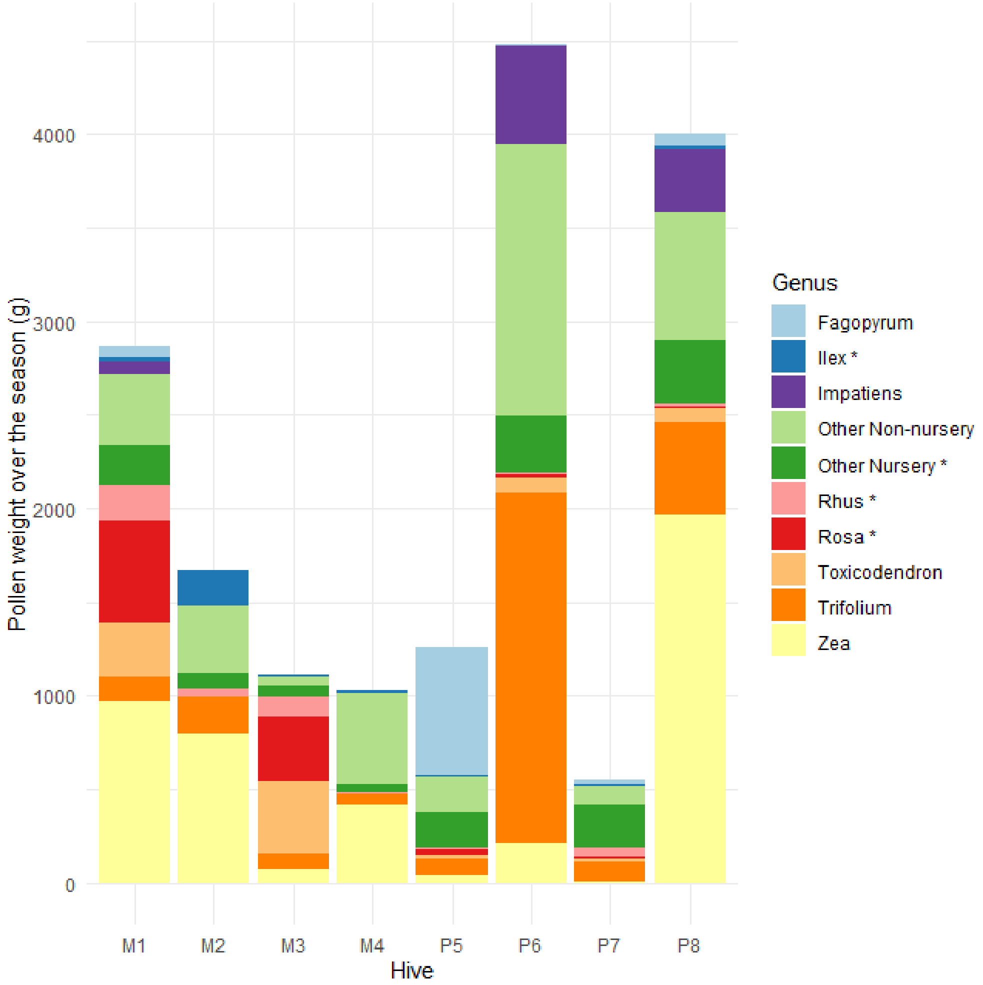

Supplemental Figure S1. Weight of trapped pollen by genus over the season for each hive in 2018. M = Monrovia Nursery, P = Prides Corner Farms, and the numbers represent the numbered hives at each site. * Indicates plant genera grown at the nursery, and “Other Nursery *” indicates the sum of all other genera grown at the nursery in addition to *Ilex*, *Rhus*, and *Rosa*. Likewise, “Other Non-nursery” indicates the sum of all genera not grown at the nursery in addition to those specifically graphed.

Supplemental Table S1. Weight of trapped pollen by genus over the season for each hive in 2018, as shown in Supplemental Fig. 1 classified by whether, or not the genus was grown at the nursery, and total and proportion of the weight of pollen from nursery or non-nursery genera.

		M1	M2	M3	M4	P5	P6	P7	P8	Total M	Total P
Nursery Sources											
	Ilex	25	191	7	16	7	1	9	18	237	35
	Rhus	194	40	109	6	8	10	54	17	349	89
	Rosa	542	0	348	2	37	19	4	13	892	73
	Other Nursery	211	78	50	39	186	301	226	335	379	1048
Total Nursery		971	309	514	63	238	330	294	383	1857	1245
Proportion from Nursery		0.34	0.19	0.46	0.06	0.19	0.07	0.54	0.10	0.28	0.12
Non-Nursery Sources											
	Fagopyrum	58	0	0	0	681	9	21	62	58	773
	Impatiens	70	1	0	0	4	527	1	335	71	866
	Toxicodendron	291	2	391	0	10	83	17	75	684	185
	Trifolium	127	202	78	55	90	1872	108	490	462	2560
	Zea	974	794	74	419	43	211	6	1969	2261	2229
	Other Non-nursery	375	362	57	488	193	1450	100	688	1281	2431
Total Non-nursery		1896	1360	599	962	1020	4152	253	3618	4817	9043
Total Pollen		2868	1669	1113	1025	1258	4482	547	4001	6674	10288

Supplemental Table S2. Weight (wt) of pollen collected by location, hive, and date, 2018.

Monrovia Nursery			Prides Corner Farms		
**Hive**	**Date**	**Wt of pollen collected**	**Hive**	**Date**	**Wt of pollen collected**
1	8-Jun	403.47	6	7-Jun	560.34
3	8-Jun	460.26	8	7-Jun	88.62
1	15-Jun	771.8	6	14-Jun	1582.48
3	15-Jun	503.97	8	14-Jun	923.27
2	22-Jun	84.87	5	21-Jun	248.70
2	29-Jun	408.13	7	21-Jun	222.21
4	29-Jun	66.03	5	27-Jun	106.01
1	6-Jul	153.26	7	27-Jun	263.89
3	6-Jul	36.88	6	5-Jul	16.59
1	13-Jul	54.96	8	5-Jul	92.81
3	13-Jul	21.07	6	12-Jul	4.92
2	20-Jul	159.09	8	12-Jul	108.37
4	20-Jul	59.67	5	19-Jul	387.62
2	27-Jul	540.01	7	19-Jul	21.30
4	27-Jul	257.1	5	26-Jul	355.60
1	3-Aug	55.29	7	26-Jul	8.39
1	10-Aug	957.8	6	2-Aug	121.39
3	10-Aug	90.55	8	2-Aug	523.20
2	17-Aug	172.12	6	9-Aug	161.12
4	17-Aug	128.02	8	9-Aug	1495.53
2	24-Aug	305.06	5	23-Aug	166.92
4	24-Aug	513.84	7	23-Aug	31.95
1	31-Aug	199.57	6	30-Aug	792.78
1	6-Sep	271.29	8	30-Aug	160.85
			6	6-Sep	1219.32
			8	6-Sep	608.21
